# Gorman revisited: nonparametric conditions for exact linear aggregation

**DOI:** 10.1007/s13209-016-0140-y

**Published:** 2016-03-05

**Authors:** Laurens Cherchye, Ian Crawford, Bram De Rock, Frederic Vermeulen

**Affiliations:** Center for Economic Studies, University of Leuven, E. Sabbelaan 53, 8500 Kortrijk, Belgium; Department of Economics, Institute for Fiscal Studies and University of Oxford, Manor Road Building, Manor Road, Oxford, OX1 3UQ United Kingdom; ECARES, Université Libre de Bruxelles and Department of Economics, University of Leuven, Avenue F. D. Roosevelt 50, CP 114, 1050 Brussels, Belgium; Department of Economics, University of Leuven, Naamsestraat 69, 3000 Leuven, Belgium

**Keywords:** Aggregation, Revealed preference, Gorman Polar Form preferences, Representative agent, C14, D11, D12, E21

## Abstract

In the tradition of Afriat (Int Econ Rev 8:67–77, 1967), Diewert (Rev Econ Stud 40:419–425, 1973) and Varian (Econometrica 50:945–972, 1982), we provide a revealed preference characterisation of exact linear aggregation. This guarantees that aggregate demand can be written as a function of prices and aggregate income alone, while abstracting from income-distributional aspects. We also establish nonparametric conditions for individual consumption to be representable in terms of Gorman Polar Form preferences. Our results are simple and complement those of Gorman (1953, 1961). We illustrate the practical usefulness of our results by means of an empirical application to a Spanish balanced microdata panel. We find strong evidence against the existence of a limited set of representative agents, which in turn seems to empirically support the need for (macroeconomic) models using a continuum of heterogeneous agents.

## Introduction

Many models in the theoretical and empirical literature on macroeconomics, international trade and industrial organisation assume, at least implicitly, that aggregate demand is invariant to changes in the income distribution over individual consumers and, hence, that aggregate demand depends only on prices and aggregate income. The best known theoretical results on this topic are probably those of Gorman (1953, 1961), who made explicit the conditions on microeconomic consumer behaviour under which aggregate demand can be written as a function of prices and aggregate income alone.^1^ Specifically, Gorman showed that exact linear aggregation is possible if and only if consumers have preferences of the Gorman Polar Form such that the corresponding linear Engel curves have common slopes. The empirical literature on consumer behaviour, however, has consistently tended to show that these conditions do not hold in practice. Lewbel and Pendakur (2009), for example, provide strong parametric evidence of nonlinear Engel curve behaviour whilst Blundell et al. (2007) consider semi- and nonparametric evidence for this non-linearity.

In this paper, we revisit the problem that Gorman addressed. We too seek necessary and sufficient conditions for exact linear aggregation. However, we do this from a rather different perspective, that of the nonparametric revealed preference tradition of Samuelson (1938, 1948), Afriat (1967), Diewert (1973) and Varian (1982). Instead of describing the restrictions on behaviour in terms of the derivatives of certain functions (the slopes of Engel curves, for example), this approach works by characterising them in terms of a finite system of inequalities involving the consumers’ observed choices only.

Finding a nonparametric equivalent to Gorman’s aggregation theorems is, of course, of a certain amount of theoretical interest, but this is not our only motivation: we are also interested in empirical implementation. In particular we are interested in whether it may be possible empirically to analyse microdata for its aggregation properties without resorting to regression analysis. Regression analysis, in the words of Daniel McFadden in his presidential address to the Econometric Society, “interposes an untidy veil between econometric analysis and the propositions of economic theory”. In fact McFadden was discussing parametric regression but nonparametric regression is not immune from the same observation. If the implications of economic theory are described in terms of the shapes of functions implied by the theory (e.g. Engel curves) then any empirical investigation of the theory requires those functions to be estimated from data. As a result, the conclusions from such an exercise necessarily rest jointly on the validity of the hypothesis at stake plus a number of crucial auxiliary statistical assumptions necessary to deliver consistent estimates of the functions of interest. This is the case whether the estimates are parametric, semi-parametric or nonparametric. For example both Lewbel and Pendakur (2009) and Blundell et al. (2007) provide evidence based on pooled cross-section data—they therefore need to make a number of carefully chosen auxiliary assumptions about the form of unobserved heterogeneity and how it enters the model in order to deliver their estimates. Both studies also need to follow an instrumental variables strategy which also brings with it a set of important identifying assumptions. Revealed preference methods do not require the identification or estimation of structural functions. Instead they involve only inequality restrictions on the observables alone. As a result they are, to a great extent, free of the need for auxiliary hypotheses. They therefore allow researchers to focus with much greater clarity on the hypothesis at the core. Furthermore, they are applicable when there are only very few observations and, hence, when statistical methods would be infeasible or uninformative.

The main contribution of this paper is twofold. Firstly, we establish the nonparametric counterparts of Gorman’s aggregation conditions. We start by providing a revealed preference characterisation of Gorman Polar Form preferences for an individual consumer. We then propose an easy-to-implement necessary and sufficient test for Gorman’s conditions for exact linear aggregation. Secondly, we demonstrate the practical usefulness of our results through an empirical investigation using a balanced microdata panel of Spanish households. Our first main conclusion here will be that we strongly reject exact linear aggregation when focusing on the set of all rational households in our sample. Our second main result is that this rejection is primarily due to heterogeneity in the marginal utility of income. To investigate this heterogeneity, we also considered the possible partitioning of our sample of households into subsets for which exact linear aggregation holds. We conducted two exercises. Firstly, we partition our sample on a standard set of observable household characteristics. Again, however, we find that exact linear aggregation is rejected for each thus defined subset of households. Secondly, we use a slight adaptation of a method introduced by Crawford and Pendakur (2013) to define a partitioning that accounts for possibly unobserved household characteristics. Essentially, this method identifies the minimal number of subsets of households such that each individual subset is consistent with exact linear aggregation. We conclude that we need a substantial number of groups (revealing unobserved heterogeneity) for the observed household consumption to be exactly aggregable.

Summarizing, given the nonparametric nature of our tests, our empirical results provide robust evidence against the existence of a representative agent. This complements the already existing empirical evidence (see, for example, Kirman 1992 and Carroll 2000), but now from a revealed preference perspective. Moreover, we also show that the existence of a limited set of representative agents seems to be a very unrealistic hypothesis. As we will discuss more in detail in Sect.  3, we interpret all this as providing empirical support to macroeconomic models working with a continuum of heterogeneous agents, such as the so-called standard incomplete markets models (see, for example, Heathcote et al. 2009).

As a final point, we remark that the revealed preference approach that we follow in this paper is completely deterministic and static in nature. First, in its pure form, it defines testable conditions that ignore any source of randomness in the data, which excludes formal statistical hypothesis testing. Importantly, however, it is possible to formally account for statistical issues by combining the exact aggregation conditions that we present below with methodological tools that have been presented in alternative revealed preference contexts. For example, it is fairly straightforward to account for measurement error by combining our results with an original proposal of Varian (1985). Next, we could account for random utility considerations by integrating our analysis with the one of McFadden and Richter (1991). To focus our discussion, we will not explicitly discuss these extensions in the current paper. Finally, as in Gorman (1953, 1961), we focus on a static framework, which makes it easier to define the concept of a representative agent. However, this of course implies that we ignore intertemporal aspects such as habit formation and/or saving decisions.^2^ To address these issues, we should for instance integrate the revealed preference characterizations on the life-cycle rational expectations hypothesis (see Browning 1989) and/or habit formation (see Crawford 2010) into our framework. We see all these points as interesting developments for follow-up research.

The remainder of this paper is structured as follows. Section 2 contains our main theoretical results, which provide a revealed preference characterisation of individual Gorman Polar Form preferences and an easy-to-implement necessary and sufficient nonparametric test for exact linear aggregation. Section 3 presents our empirical application. Section 4 concludes.

## Exact linear aggregation: a nonparametric characterisation

In this section, we start by briefly reviewing the revealed preference conditions for rational consumption behaviour of individual consumers in terms of the well-known Afriat inequalities. Then, we investigate the conditions needed to guarantee exact linear aggregation, i.e. aggregate demand only depends on aggregate income and is not affected by how the income is actually distributed across consumers. From the functional derivative-based literature, we know that this independence result applies if and only if consumers have preferences of the Gorman Polar Form and linear Engel curves with common slopes. From this, we can define our revealed preference counterparts of these Gorman-type aggregation conditions. We will proceed in two main steps. Firstly, we derive a revealed preference characterisation of individual preferences of the Gorman Polar Form. Subsequently, we present the revealed preference version of Gorman’s aggregation conditions stated above. This will define an easy-to-apply linear test for exact linear aggregation.

Before moving on, it is worth pointing out the well-established fact that the Gorman Polar Form does not necessarily give rise to well-behaved preferences in all parts of the quantity-space: in general, well-behaved preferences only apply to a limited range of possible income values. For instance, for some income values, the linear Engel curves may lead to negative consumption or cross with each other. To avoid such problems, Gorman Polar Form preferences are usually defined subject to bounds on possible income levels.^3^ To keep the exposition simple, our following analysis only considers income values that lie within such income ranges and, thus, we will not explicitly consider income bounds in our exposition. But it should be kept in mind that our following characterisations of Gorman Polar Form preferences and exact linear aggregation are essentially “local” in that they apply to sufficiently small changes in the incomes of individual consumers. In our proof of Theorem 2, we indicate how the relevant income bounds can be computed in empirical applications [see our discussion of program (3) in Appendix 1].

### Gorman Polar Form preferences

Suppose that we have a balanced microdata panel of consumers indexed by $$ h=1,...,H$$ observed over a number of periods indexed $$t=1,...,T$$. For each consumer *h* we observe non-negative consumption quantities $$\mathbf {q} _{t}^{h}\in \mathbb {R}_{+}^{K}$$, where *K* is the number of goods. Following Gorman (1953), we make the classical assumption that the law of one price holds (i.e. all households face the same price) and that prices are strictly positive *K*-vectors ($$\mathbf {p}_{t}\in \mathbb {R}_{++}^{K}$$). We will denote these microdata by $$\left\{ \mathbf {p}_{t},\mathbf {q}_{t}^{h}\right\} _{t\in \tau }^{h\in \eta }$$, with $$\eta =\left\{ 1,...,H\right\} $$ and $$\tau =\left\{ 1,...,T\right\} $$ being the index sets for consumers and periods, respectively. We will use $$\mathbf {Q}_{t}=\sum _{h\in \eta }\mathbf {q} _{t}^{h} $$ to denote the aggregate demand vector in period *t*, so that the macrodata are $$\left\{ \mathbf {p}_{t},\mathbf {Q}_{t}\right\} _{t\in \tau }$$. Aggregate income is denoted by $$Y_{t}$$ and is equal to $$\mathbf {p} _{t}^{\prime }\sum _{h\in \eta }\mathbf {q}_{t}^{h}=\mathbf {p}_{t}^{\prime } \mathbf {Q}_{t}$$.

*Individual rationality.*

We will assume that all the consumers are rational in the sense that observed demand results from the maximisation of a well-behaved utility function subject to an individual budget constraint. Throughout, we will assume that utility functions are well-behaved (i.e. monotonically increasing, concave and continuous). We can formally define individual rationality as follows:

#### **Definition 1**

(Individual rationalisation) A well-behaved utility function $u^{h}$ provides an *individual rationalisation *of the data $\left\{ \mathbf {p }_{t},\mathbf {q}_{t}^{h}\right\} _{t\in \tau }$ if for each observation $t\in \tau$ we have $u^{h}\left( \mathbf {q}_{t}^{h}\right) \ge u^{h}\left( \mathbf {q}\right)$ for all $$\mathbf {q}\ $$with $\mathbf {p}_{t}^{\prime } \mathbf {q}\le \mathbf {p}_{t}^{\prime }\mathbf {q}_{t}^{h}$.

For our following discussion it is useful to be more specific about the empirical content of individual rationalisation. A core result in the revealed preference approach to demand is that there exists a utility function that provides an individual rationalisation of the data $\left\{ \mathbf {p}_{t},\mathbf {q}_{t}^{h}\right\} _{t\in \tau }$ if and only if the data satisfy the well-known Afriat inequalities. This is formally captured by Afriat’s Theorem (Varian 1982; based on Afriat 1967):

#### **Theorem 1**

(Afriat’s Theorem) The following statements are equivalent:

1. There exists an individual rationalisation of the data $\left\{ \mathbf {p}_{t},\mathbf {q}_{t}^{h}\right\} _{t\in \tau }$.
2. For all $t\in \tau$, there exist numbers $u_{t}^{h}\in \mathbb {R} _{+}$ and $\beta _{t}^{h}\in \mathbb {R} _{++}$ that satisfy the *Afriat inequalities*, i.e. for all $s,t\in \tau$:
  $$\begin{aligned} u_{s}^{h}\le u_{t}^{h}+\beta _{t}^{h}\mathbf {p}_{t}^{\prime }\left( \mathbf { q}_{s}^{h}-\mathbf {q}_{t}^{h}\right) \text {.} \end{aligned}$$

The theorem thus implies that any data set $$\left\{ \mathbf {p}_{t},\mathbf {q} _{t}^{h}\right\} _{t\in \tau }$$ that can be rationalised by a well-behaved utility function needs to satisfy the Afriat inequalities. These Afriat inequalities are linear inequalities that are expressed in the unknowns $$ u_{t}^{h}$$ and $$\beta _{t}^{h}$$ and that can easily be verified.^4^ They also allow us to obtain an explicit construction of the utility levels and the marginal utility of income associated with each observation *t*: they define a utility level $$u_{t}^{h}$$ and a marginal utility of income $$ \beta _{t}^{h}$$ (associated with the observed income $$\mathbf {p}_{t}^{\prime }\mathbf {q}_{t}^{h}$$) for each observed $$\mathbf {q}_{t}^{h}$$. As has been demonstrated by Varian (1982), and later by Blundell, Browning and Crawford (2003, 2008) and Blundell et al. (2015), the above insights can be used to formally evaluate policy reforms in terms of individual welfare by computing, for instance, bounds on equivalent and compensating variations.

*Gorman Polar Form preferences.*

We next define what it means for the data of an individual consumer to be rationalisable with the Gorman Polar Form. The Gorman Polar Form is usually defined in terms of an indirect utility function $w^{h}$. Let $y^{h}$ represent the income of consumer *h*. The indirect utility function $w^{h}$ is connected with the direct utility function $u^{h}$ in the following way:

$$\begin{aligned} w^{h}\left( \mathbf {p},y^{h}\right) =\max _{\mathbf {q}^{h}}\{u^{h}\left( \mathbf {q}^{h}\right) |\mathbf {p}^{\prime }\mathbf {q}^{h}\le y^{h}\}. \end{aligned}$$

We can now state the next definition.

#### **Definition 2**

(*Gorman Polar Form rationalisation*) The data $$\left\{ \mathbf {p}_{t},\mathbf {q}_{t}^{h}\right\} _{t\in \tau }$$ are rationalisable by the Gorman Polar Form if there exists a utility function $$u^{h}$$ that provides an individual rationalisation of the data and if there exists an associated indirect utility function $$w^{h}( \mathbf {p},y^{h})=\frac{y^{h}-a^{h}(\mathbf {p})}{b^{h}(\mathbf {p})}$$, with $$ a^{h}(\mathbf {p})\in \mathbb {R}$$ and $$b^{h}(\mathbf {p})\in \mathbb {R}_{++}$$ for all $$\mathbf {p}$$ and the functions $$a^{h} $$ and $$b^{h}$$ homogeneous of degree 1.

In this definition, the price index $a^{h}(\mathbf {p})$ is often interpreted as subsistence expenditure—although this interpretation is not always valid (see Pollak 1971, p. 403, fn. 4)—while the price index $b^{h}( \mathbf {p})$ is interpreted as the inverse of the marginal utility of income.

We can then state the characterisation.^5^

#### **Theorem 2**

The following statements are equivalent:

(2.A). The data $\left\{ \mathbf {p}_{t},\mathbf {q}_{t}^{h}\right\} _{t\in \tau }$ are rationalisable by the Gorman Polar Form.
(2.B). For all $t\in \tau ,$ there exist numbers $w_{t}^{h}\in \mathbb {R} _{+}$, $a_{t}^{h}\in \mathbb {R}$ and $b_{t}^{h}\in \mathbb {R}_{++}$ such that for all $s,t\in \tau$:
  2.B.1 $$\begin{aligned}&w_{s}^{h}\le w_{t}^{h}+\frac{1}{b_{t}^{h}}\mathbf {p}_{t}^{\prime }\left( \mathbf {q}_{s}^{h}-\mathbf {q}_{t}^{h}\right) {,} \end{aligned}$$
  2.B.2 $$\begin{aligned}&w_{t}^{h}=\ \frac{\left( \mathbf {p}_{t}^{\prime }\mathbf {q}_{t}^{h}\right) -a_{t}^{h}}{b_{t}^{h}}, \end{aligned}$$
  2.B.3 $$\begin{aligned}&a_{t}^{h}=\delta a_{s}^{h}\text { and }b_{t}^{h}=\delta b_{s}^{h}\text { if } \mathbf {p}_{t}=\delta \mathbf {p}_{s}\text { for }\delta \in \mathbb {R}_{++}. \end{aligned}$$

Similar to Theorem 1, the numbers in this result have certain structural interpretations. Condition $\left( 2.B.1\right)$, for example, is an Afriat inequality which allows us to construct a utility level $\left( w_{t}^{h}\right)$ and marginal utility of income $\left( 1/b_{t}^{h}\right)$ for each observation *t*. We can interpret every $w_{t}^{h}$ as an indirect utility value (the function value $w^{h}(\mathbf {p},y^{h})$ in Definition 2, which equals the utility value $u^{h}\left( \mathbf { q}^{h}\right)$ under rational consumer behaviour). Condition $\left( 2.B.2\right)$ then states the Gorman Polar Form restriction, with the numbers $a_{t}^{h}$ and $b_{t}^{h}$ corresponding to the price indices $a^{h}(\mathbf {p})$ and $b^{h}(\mathbf {p})$ in Definition 2 evaluated at $\mathbf {p}_{t}$.^6^ Condition $\left( 2.B.3\right)$, finally, imposes homogeneity of these price indices.

Two final notes are in order. Firstly, the Gorman Polar Form characterisation in Theorem 2 is nonlinear in $$a_{t}^{h}$$ and $$ b_{t}^{h}$$. However, in our proof of Theorem 2 we show that it can be equivalently expressed in linear form. This makes it computationally very convenient. Secondly, in the absence of proportional price movements, Gorman Polar Form preferences provide no additional restrictions over and above the standard Afriat inequalities stated in Theorem 1.^7^ In other words, Gorman Polar Form preferences and rational preferences are nonparametrically (in the revealed preference sense) equivalent: for data in which proportional price movements are not observed their empirical implications are identical.^8^

### Exact linear aggregation

We can now use these insights to provide the revealed preference counterpart of Gorman’s conditions for exact linear aggregation. As stressed above, exact linear aggregation implies that aggregate demand only depends on prices and aggregate income and is thus independent of the income distribution. Formally, this implies that aggregate demand can be written in the simple form $\mathbf {Q}=\mathbf {g}\left( \mathbf {p,}Y\right)$, where $\mathbf {g}\left( \mathbf {p,}Y\right)$ is a vector-valued demand equation that only depends on aggregate income $Y=\sum _{h\in \eta }y^{h}$ and prices $\mathbf {p}$. In other words, any income distribution of a given *Y* gives rise to the same aggregate demands $\mathbf {Q}$.

Gorman proved that such exact linear aggregation holds if and only if consumers’ preferences are of the Gorman Polar Form with common slopes for the linear Engel curves. This is translated formally in the following definition:

#### **Definition 3**

*(Exact linear aggregation)* The data $\left\{ \mathbf {p}_{t},\mathbf {q} _{t}^{h}\right\} _{t\in \tau }^{h\in \eta }$ satisfy the conditions for exact linear aggregation if, for each $h\in \eta$, the data are rationalisable by the Gorman Polar Form and, moreover, the associated indirect utility functions are given by $w^{h}(\mathbf {p},y^{h})=\frac{ y^{h}-a^{h}(\mathbf {p})}{b(\mathbf {p})}$, i.e. we have $b^{h}(\mathbf {p})=b( \mathbf {p})$.

In terms of Definition 2, exact linear aggregation requires a common $b\left( \mathbf {p}\right)$ index for all consumers (i.e. $b^{h}\left( \mathbf {p}\right) =b\left( \mathbf {p}\right)$ for all *h*). The idea is that the marginal utility of income must be independent of income variations across consumers but can vary with prices. Using Theorem 2, we get the following characterisation of exact linear aggregation in revealed preference terms.

#### **Theorem 3**

The following statements are equivalent for the data $\left\{ \mathbf {p}_{t},\mathbf {q}_{t}^{h}\right\} _{t\in \tau }^{h\in \eta }$ : (3.A). The data $\left\{ \mathbf {p}_{t},\mathbf {q}_{t}^{h}\right\} _{t\in \tau }^{h\in \eta }$ satisfy the conditions for exact linear aggregation.

(3.B). For all $t\in \tau$ and $h\in \eta$, there exist numbers $w_{t}^{h}\in \mathbb {R}_{+}$, $a_{t}^{h}\in \mathbb {R}$ and $b_{t}\in \mathbb {R}_{++}$ such that for all $s,t\in \tau$:
  3.B.1 $$\begin{aligned}&w_{s}^{h}\le w_{t}^{h}+\frac{1}{b_{t}}\mathbf {p}_{t}^{\prime }\left( \mathbf {q}_{s}^{h}-\mathbf {q}_{t}^{h}\right) {,} \end{aligned}$$
  3.B.2 $$\begin{aligned}&w_{t}^{h}=\ \frac{\left( \mathbf {p}_{t}^{\prime }\mathbf {q}_{t}^{h}\right) -a_{t}^{h}}{b_{t}}, \end{aligned}$$
  3.B.3 $$\begin{aligned}&a_{t}^{h}=\delta a_{s}^{h}\quad \text { and }\quad b_{t}=\delta b_{s}\text { if } \ \mathbf { p}_{t}=\delta \mathbf {p}_{s}\quad \text { for } \ \delta \in \mathbb {R}_{++}. \end{aligned}$$

As compared to Theorem 2, the key requirement is that the Afriat number $$b_{t}$$ is common across consumers who face the same prices (i.e. $$ b_{t}^{h}=b_{t}$$ for all *h*). Referring to Definition 2, this effectively imposes Gorman Polar Form preferences with a common $$b\left( \mathbf {p}\right) $$ index for all consumers. We note, finally, that our characterisation in Theorem 3 can be linearised in a directly similar way as our earlier characterisation in Theorem 2. As such, it implies an easy-to-apply linear test for exact linear aggregation.

Interestingly, the characterisation in Theorem 3 also generalises several special cases that generate the same independence of the income distribution. Two important examples are Varian’s (1983) revealed preference characterisation of identical homothetic preferences (where $a^{h}\left( \mathbf {p}\right) =0$ in Definition 2) and Brown and Calsamiglia’s (2007) revealed preference characterisation of quasi-linear preferences (where $a^{h}\left( \mathbf {p}\right) =-p^{i}\phi \left( \mathbf { p}\right)$ and $b^{h}\left( \mathbf {p}\right) =p^{i}$, with $p^{i}$ the price of the numeraire and $\phi$ a function that is homogeneous of degree one).

Finally, from the characterisation in Theorem 3 we obtain that, if observed price movements are nonproportional, then a necessary and sufficient condition for exact linear aggregation is that each consumer satisfies the standard Afriat inequalities with a common marginal utility of income. This is formally stated in the following result:

#### **Corollary 1**

If for all $s,t\in \tau$ and $\delta \in \mathbb {R} _{++}$ we have that $\mathbf {p}_{t}\ne \delta \mathbf {p}_{s}$, then the following statements are equivalent:

(A). The data $\left\{ \mathbf {p}_{t},\mathbf {q}_{t}^{h}\right\} _{t\in \tau }^{h\in \eta }$ satisfy the conditions for exact linear aggregation.
(B). For all $t\in \tau$ and $h\in \eta$, there exist numbers $w_{t}^{h}\in \mathbb {R}_{+}$ and $b_{t}\in \mathbb {R}_{++}$ such that for all $s,t\in \tau$:
  $$\begin{aligned} w_{s}^{h}\le w_{t}^{h}+\frac{1}{b_{t}}\mathbf {p}_{t}^{\prime }\left( \mathbf {q}_{s}^{h}-\mathbf {q}_{t}^{h}\right) {.} \end{aligned}$$

## An application

In the previous section we established the revealed preference conditions for exact linear aggregation. Importantly, our characterisation can be linearised in unknowns, which makes it easily testable. We will next illustrate our revealed preference based aggregation results by means of an empirical application. Here, it is worth to recall from our discussion in the Introduction that revealed preference methods are intrinsically “nonparametric”: in contrast to the more standard functional-derivative based methods, they do not need auxiliary parametric or statistical assumptions. As such, this empirical revealed preference analysis should thus lead to robust conclusions.

### The data

The data we use are drawn from the Spanish Continuous Family Expenditure Survey (ECPF). This is one of the few surveys with detailed expenditure information for a panel of households. The ECPF is a quarterly budget survey of Spanish households which interviews about 3200 households every quarter. We focus on a subsample of couples (with or without children), in which the husband is in full-time employment in a non-agricultural activity while the wife is out of the labour force.^9^ Note that we assume that household preferences over consumption are separable from labour supply decisions. Therefore we want to minimise the impact of this assumption by keeping the employment status constant over the whole observation period.

Given the construction of the ECPF, households can be interviewed for up to eight consecutive quarters. However, our sample would be rather small if we would focus on those households observed for a full eight periods. Therefore, we have drawn a balanced panel of 342 households which are observed five consecutive quarters in order to balance the desire for a reasonable number of observations both across households and time. This implies that we (only) assume stable preferences over a period of five quarters.^10^ Finally, in what follows, we focus on a set of 15 nondurable commodity groups.^11^ We note that the movements in the observed price vectors are nonproportional.

Our following analysis proceeds in two steps. First we check, individual household by individual household, whether observed behaviour is rationalisable by the Gorman Polar Form, albeit with heterogeneous $b^{h}( \mathbf {p})$ indices for the different households. In the second step we then pool the data across households to investigate the conditions for exact linear aggregation.

### Rationalisability by the Gorman Polar Form

For every household in our sample we test whether their behaviour is rationalisable by preferences of the Gorman Polar Form. There are two important points to note about this procedure. Firstly, since there are no proportional price movements observed in the data, this is equivalent to testing whether the data satisfy the standard Afriat inequalities (see our discussion at the end of Sect.  2.1).^12^ Secondly, we deal with each household individually and so we allow for complete preference heterogeneity within the Gorman class: households may differ with respect to whether they are rationalisable (by this class) and the precise form of their preferences within this class. The results are presented in Table 1.

**Table 1:** Tests of Gorman Polar Form rationalisation

	Pass	Fail
N	326	16
Proportion	$\underset{\left( 0.011\right) }{0.953}$	$\underset{\left( 0.053\right) }{0.047}$

It turns out that the behaviour of 95 % of the households in our data is rationalisable by preferences of the Gorman Polar Form. A little under 5 % of the data (16 households) are not rationalisable by well-behaved preferences at all. Given the estimates of these proportions, we can also compute the corresponding standard errors of this Bernoulli experiment, which are reported in parentheses. We see that the small number of failures we observe in this sample is subject to sampling variation: it might easily be the case that another similarly sized random sample of households from this population would contain no failures at all. Since it is a necessary condition for exact linear aggregation that individual households act as if they are utility maximisers, we therefore do not include these 16 households in the further analysis.^13^

### Testing for exact linear aggregation

We now turn to the main question - given microdata with rational agents does the observed behaviour support exact linear aggregation? In other words, is their aggregate demand independent of the income distribution? To do this we check the condition that is given in Theorem 3. At this point the test requires pooling across households so that we can investigate the commonality of the marginal utility of incomes within the sample. Specifically, we need to check for the data pooled across households whether there exist numbers $$w_{t}^{h}\in \mathbb {R}_{+}$$ for $$h=1,...,326$$ and $$ t=1,...,5$$ and $$b_{t}\in \mathbb {R}_{++}$$ for $$t=1,...,5$$ such that,

$$\begin{aligned} w_{s}^{h}\le w_{t}^{h}+\frac{1}{b_{t}}\mathbf {p}_{t}^{\prime }\left( \mathbf {q}_{s}^{h}-\mathbf {q}_{t}^{h}\right) , \end{aligned}$$

for all observations $s,t\in \tau$ and all households. To do this we use phase one of the simplex method linear programming algorithm, which efficiently determines whether or not a system of linear inequalities has a basic feasible solution. We find that this condition is rejected. Despite the fact that these households all satisfy the necessary condition (Gorman Polar Form preferences), and despite the very flexible nature of revealed preference tests, it seems that the additional restriction required for aggregation (that within a period all households have the same $b_{t}$ parameter) is too much: data cannot bear the weight of the theory required for exact linear aggregation.

### Preference heterogeneity

Given that the behaviour of our remaining households is precisely consistent with the idea that they have preferences of the Gorman Polar Form albeit with different preferences within that class, we now turn to the issue of heterogeneity. The focus is on the heterogeneity which is relevant for aggregation, namely heterogeneity with respect to the marginal utility of income.

*Partitioning on observables.*

To the extent that heterogeneity in the $b_{t}$ parameter might be driven by observables, stratification is a flexible and fully nonparametric way in which to allow for this –the idea being that exact linear aggregation might be valid when applied to sub-groups of demographically similar households, even though when applied to the data *in toto* it is rejected. To investigate this further we allocated the data to relatively homogeneous groups on the basis of observables such as their age profiles, schooling level, household size and number of children. This resulted in 52 groups of which 34 groups contain more than one household (see Appendix 2 for the frequency distribution of the group sizes). We then test the conditions for exact linear aggregation once more but this time *within* each of these groups. Although the number of different households within a group can be as small as two, as long as there is more than one household the conditions for exact linear aggregation always fail. This despite the fact that the strength of revealed preference tests in general (weakly) increases with the number of observations, so that reducing the number of households involved in a test, by considering only those with similar observables, should make it easier to rationalise behaviour.

*Partitioning on unobservables.*

We also consider a second partitioning exercise. As before, the idea is to partition our set of 326 rational households into a number of subsets, such that each subset contains households which, together, are exactly linearly aggregable. Instead of partitioning on observables we use an algorithm which searches for the most parsimonious grouping, i.e. one that minimises the number of groups required to exclusively and exhaustively partition the data into groups within which behaviour is aggregable. Of course, the only way to determine this precisely is to form the set of all subsets of the data (and there are 2$^{326}$ of these) and to check all of them.

Since this is computationally infeasible we adapt an easy to implement algorithm developed by Crawford and Pendakur (2013). These authors present a revealed preference-based method that bounds the minimal partition of consumer microdata into a set of preference types such that each subset is perfectly rationalisable by standard utility maximization. This provides a simple, non-parametric and theory-driven way of investigating unobserved preference heterogeneity in empirical data. We adapt this algorithm to our setting (i.e. replace the revealed preference conditions for utility maximization by the ones of exact linear aggregation) and as such we can calculate bounds on the minimal number of aggregable groups.

We find that the fewest number of groups we need to rationalise the data is somewhere between 90 and 103. Clearly $52\notin \left[ 90,103\right]$ and this is why our attempt to rationalise the data on the previous demographics-based partition failed. On average, there are fewer than 4 households per aggregable subset, and the largest group we were able to construct consisted of 19 households. Essentially, this outcome says that we need a substantial number of groups (including many singletons) to rationalise our data in terms of exact linear aggregation.

*Interpretation.*

Generally, our results appear to indicate that heterogeneity in the marginal utility of income is both essentially idiosyncratic and economically meaningful in the sense that it is sufficient, firstly, to be easily detectable with a nonparametric/revealed preference test and, secondly, to prevent exact linear aggregation from going through.

It is interesting to interpret this finding in the light of recent developments in the macroeconomics literature. The advent of more powerful computers and improved numerical methods did not only give birth to the field of microeconometrics, but it also allowed macroeconomists to shift attention towards rich heterogeneous agents models. Nowadays, one of the main workhorse models for studying heterogeneity in macroeconomics is what Heathcote et al. (2009) call the “standard incomplete markets” (SIM) model (see also Ríos-Rull 1995; Ljungqvist and Sargent 2004, and Krusell and Smith 2006). The SIM model is characterised by a continuum of individuals (households), who have different preferences and who differ with respect to characteristics like productivity or health status. These individuals then are faced with independent uninsurable shocks in their endowments, which lead to behavioural changes at the micro level and, ultimately, also at the macro level. By construction, our nonparametric method allows for considerably more heterogeneity then in the standard SIM model. However, even in our minimalisatic set-up, we still reject exactly aggregable behavior unless we explicitly account for (unobservable) heterogeneity across households. In our opinion, we may take our results as providing specific empirical support for using (SIM-type) macroeconomic models with a continuum of heterogenous households.

Related to this, our results can also be interpreted as revealed preference evidence against the existence of a representative agent.^14^ If the conditions for linear exact aggregation are satisfied, then there exists a representative agent for which the aggregate demand can be modelled as the outcome of rational, maximising behaviour given prices and aggregate income. Importantly, this representative agent also allows for normative conclusions: the agent’s preferences can properly be represented by an aggregate social welfare function.^15^ As such, our empirical results also complement the overwhelming evidence against the existence such a representative agent (see, for example, Kirman 1992, and Carroll 2000). More specifically, they add that even the existence of a limited set of representative agents seems to be a very unrealistic hypothesis. As indicated above, because our test is intrinsically nonparametric, it provides robust evidence in support of this conclusion.

## Conclusion

Many economic models use the assumption that aggregate demand depends solely on prices and aggregate income and, thus, abstract from income-distributional effects. Although the conditions for the existence of such a distribution-independent aggregation have been argued to be demanding, it is fair to say that existing evidence is solely based on Gorman’s well-known exact linear aggregation results within a functional-derivative based framework. To test Gorman’s conditions for exact linear aggregation (which boil down to consumers having preferences of the Gorman Polar Form with an equal marginal utility of income), one needs to make many additional assumptions to bring these conditions to the data.

In this paper, we revisited the problem of exact linear aggregation by bringing in tools from the revealed preference literature. These tools are based solely on the data at hand and do not need any additional parametric or statistical assumptions. As such, they allow for robustly analysing the empirical validity of exact linear aggregation. In addition to a few interesting and rather important side results (like a revealed preference characterisation of Gorman Polar Form preferences for an individual consumer), we proposed a revealed preference test for exact linear aggregation. Interestingly, the test is linear and thus easy-to-apply in practice.

We demonstrated the practical usefulness of our revealed preference characterisation by means of an empirical application to a Spanish balanced microdata panel. Our main conclusion is that we could not find any evidence suggesting the existence of a limited set of household types for which aggregate demand can be modelled as independent of the income distribution. That is, our conditions for exact linear aggregation are not satisfied for our sample, and the same result holds even when considering small groups of households defined in terms of observable characteristics. Moreover, even an algorithmic approach designed to group the data as efficiently as possible into aggregable groups failed to find a parsimonious grouping. We may interpret these results as providing empirical support for (e.g. SIM) macroeconomic models that are based on a continuum of consumers rather than a limited set of representative agents.

## Appendix 1

### *Proof of Theorem 2*

As a preliminary step, we provide an equivalent linear formulation of the conditions in $$\left( 2.B\right) .$$ Let $$\alpha _{t}^{h}=$$$$ -a_{t}^{h}/b_{t}^{h}$$ and $$\beta _{t}^{h}=$$$$1/b_{t}^{h}$$. Then we get the following linear reformulations of the conditions $$\left( 2.B.1\right) \hbox {--}\left( 2.B.3\right) $$:

2.B.1' $$\begin{aligned} w_{s}^{h}\le w_{t}^{h}+\beta _{t}^{h}\mathbf {p}_{t}^{\prime }\left( \mathbf { q}_{s}^{h}-\mathbf {q}_{t}^{h}\right) {,} \end{aligned}$$

2.B.2' $$\begin{aligned} w_{t}^{h}=\alpha _{t}^{h}+\beta _{t}^{h}\left( \mathbf {p}_{t}^{\prime } \mathbf {q}_{t}^{h}\right) , \end{aligned}$$

2.B.3' $$\begin{aligned} \alpha _{t}^{h}=\alpha _{s}^{h}\quad \text {and}\quad \beta _{t}^{h}=\beta _{s}^{h}/\delta \quad \text { if }\mathbf {p}_{t}=\delta \mathbf {p}_{s}\quad \text { for } \ \delta \in \mathbb {R}_{++}. \end{aligned}$$

$$\left( 2.A\right) \Rightarrow \left( 2.B\right) :\ $$Condition (2.B.1’) readily follows Theorem 1 for a utility function $$ u^{h}$$ that rationalises the data $$\left\{ \mathbf {p}_{t},\mathbf {q} _{t}^{h}\right\} _{t\in \tau }$$. Then, we can use $$w_{t}^{h}=\max _{\mathbf { q}}\{u^{h}\left( \mathbf {q}\right) |\mathbf {p}_{t}^{\prime }\mathbf {q}\le \mathbf {p}_{t}^{\prime }\mathbf {q}_{t}^{h}\}$$ (using $$\mathbf {p}_{t}^{\prime }\mathbf {q}_{t}^{h}=y_{t}^{h}$$). Given this, Definition 2 directly implies (2.B.2’) and (2.B.3’) when using $$\alpha _{t}=$$$$ -a^{h}(\mathbf {p}_{t})/b^{h}(\mathbf {p}_{t})$$ and $$\beta _{t}^{h}=$$$$1/b^{h}( \mathbf {p}_{t})$$.

$$\left( 2.B\right) \Rightarrow \left( 2.A\right) :\ $$Consider

$$\begin{aligned} u^{h}\left( \mathbf {q}\right) =\min _{t}\text { }[w_{t}^{h}+\beta _{t}^{h} \mathbf {p}_{t}^{\prime }\left( \mathbf {q}-\mathbf {q}_{t}^{h}\right) ]{.} \end{aligned}$$

Varian (1982) has shown that this utility function rationalises the data $\left\{ \mathbf {p}_{t},\mathbf {q}_{t}^{h}\right\} _{t\in \tau }$. Using (2.B.2’), we have

1 $$\begin{aligned} u^{h}\left( \mathbf {q}\right) =\min _{t}[\alpha _{t}^{h}+\beta _{t}^{h} \mathbf {p}_{t}^{\prime }\mathbf {q}]{.} \end{aligned}$$

Let us then verify whether the function $$u^{h}$$ satisfies Definition . Consider some arbitrary prices $$\mathbf {p}_{0}$$ and income $$ y_{0}^{h}$$. As a preliminary step, we recall that

$$\begin{aligned} w^{h}(\mathbf {p}_{0},y_{0}^{h})=\max _{\mathbf {q}}\{u^{h}\left( \mathbf {q} \right) |\mathbf {p}_{0}^{\prime }\mathbf {q}\le y_{0}^{h}\}. \end{aligned}$$

Thus, using (1), we get

$$\begin{aligned} w^{h}(\mathbf {p}_{0},y_{0}^{h})=\max _{\mathbf {q}}\left\{ \min _{t}[\alpha _{t}^{h}+\beta _{t}^{h}\mathbf {p}_{t}^{\prime }\mathbf {q}]|\mathbf {p} _{0}^{\prime }\mathbf {q}\le y_{0}^{h}\right\} \text {{.}} \end{aligned}$$

We can equivalently state this as

$$\begin{aligned} w^{h}(\mathbf {p}_{0},y_{0}^{h})=\max _{w,\mathbf {q}}\left\{ w|w\le \alpha _{t}^{h}+\beta _{t}^{h}\mathbf {p}_{t}^{\prime }\mathbf {q}\text { }\left( \forall t\in \tau \right) \mathbf {,}\text { }\mathbf {p}_{0}^{\prime }\mathbf {q }\le y_{0}^{h}\right\} , \end{aligned}$$

which obtains the linear program

2 $$\begin{aligned}&w^{h}(\mathbf {p}_{0},y_{0}^{h})=\max _{w\in \mathbb {R},\mathbf {q}\in \mathbb {R }_{+}^{N}}w \nonumber \\&\text {s.t.} \\&w-\beta _{t}^{h}\mathbf {p}_{t}^{\prime }\mathbf {q}\le \alpha _{t}^{h}\text { }\left( \forall t\in \tau \right) , \nonumber \\&\mathbf {p}_{0}^{\prime }\mathbf {q}\le y_{0}^{h}.\nonumber \end{aligned}$$

The dual linear program is given as

3 $$\begin{aligned}&w^{h}(\mathbf {p}_{0},y_{0}^{h})=\min _{\theta _{t}\in \mathbb {R}_{+}\text { } \left( t\in \tau \right) ,\lambda \in \mathbb {R}_{+}}\sum \nolimits _{t=1}^{T}\alpha _{t}^{h}\theta _{t}+\lambda y_{0}^{h} \nonumber \\&\text {s.t.} \nonumber \\&\sum \nolimits _{t=1}^{T}\theta _{t}=1, \nonumber \\&-\sum \nolimits _{t=1}^{T}\theta _{t}\beta _{t}^{h}\mathbf {p}_{t}+\lambda \mathbf {p}_{0}\ge 0. \end{aligned}$$

Let $$\theta _{t}^{*}\ \left( t\in \tau \right) $$ and $$\lambda ^{*}$$ define the optimum of program (3). In general, these optimal values are independent of $$y_{0}^{h}$$ when $$y_{0}^{h}$$ respects conditions that limit the domain of $$y_{0}^{h}$$. In practice, the boundary values for $$ y_{0}^{h}$$ can be determined by standard methodology for sensitivity analysis of linear programming. (Technically, these bounds will correspond to the range of $$y_{0}^{h}$$ (as the objective coefficient of $$\lambda $$) for which the optimal basic feasible solution of the linear program (3) remains constant.) These domain conditions parallel the usual conditions that apply to indirect utility functions representing Gorman Polar Form preferences; see our discussion following Definition 2 in the main text.

Thus, because the solution of the problem (3) is independent of $$ y_{0}^{h}$$ (under the stated domain conditions), we conclude that the function $$w^{h}$$ in (3) satisfies the requirement in Definition 2 for

$$\begin{aligned} \lambda ^{*}=1/b^{h}(\mathbf {p}_{0})\quad \text { and }\quad -a^{h}(\mathbf {p} _{0})/b^{h}(\mathbf {p}_{0})=\sum \nolimits _{t}\theta _{t}^{*}\alpha _{t}^{h}\text {.} \end{aligned}$$

Specifically, for $w^{*}$ the optimal value of linear program (3 ) [or, equivalently, (2)], we get

$$\begin{aligned} w^{h}(\mathbf {p}_{0},y_{0}^{h})= & {} w^{*} \\= & {} \lambda ^{*}y_{0}^{h}+\sum \nolimits _{t=1}^{T}\theta _{t}^{*}\alpha _{t}^{h} \\= & {} \frac{y_{0}^{h}-a^{h}(\mathbf {p}_{0})}{b^{h}(\mathbf {p}_{0})}. \end{aligned}$$

Inspection of problems (2) and (3) reveals that the price indices $a^{h}$ and $b^{h}$ are linearly homogenous of degree 1 (if again the same income domain conditions hold).$\square$

### *Proof of Theorem 3*

This follows from Theorem 2 (i.e. each household is rationalisable by the Gorman Polar Form) and the result of Gorman (i.e. the marginal utility of income is household independent, which is captured by the common $$ b_{t}$$ (i.e. $$b_{t}^{h}=b_{t}$$ for all *h*)). $$\square $$

### *Proof of Corollary 1*

Condition (3.*B*.3) in Theorem 2 is void if $\mathbf {p}_{t}\ne \delta \mathbf {p}_{s}$ ($\delta \in \mathbb {R}_{++}$) for all $s,t\in \tau$. Moreover, any solution for condition (3.*B*.1) automatically satisfies condition (3.*B*.2). Indeed, take $a_{t}^{h}$ equal to $\mathbf {p} _{t}^{\prime }\mathbf {q}_{t}^{h}-w_{t}^{h}b_{t}^{h}$. As such, rationalisability under exact linear aggregation only requires consistency with the condition (3.*B*.1). We remark that this equivalence result is only local, as already discussed in the beginning of Sect. 2.$\square$

## Appendix 2

See Table 2.

**Table 2:** Household sub-groups

Group size	Frequency
1	18
2	6
3	4
4	6
5	1
6	2
7	3
8	2
9	1
10	2
11	1
14	1
20	1
22	1
24	1
35	1
51	1

## Footnotes

1 Exact linear aggregation is to be distinguished from exact nonlinear aggregation, where aggregate demand is a function of some representative level of aggregate income, which itself can be a function of the distribution of income over the individuals. See, for example, Muellbauer (1975, 1976) and Jerison (1994) for more discussion.

2 See, for instance, Carrasco et al. (2005), who present empirical evidence for the presence of habits for the same Spanish panel data.

3 See, for example, Pollak (1971) and Blackorby et al. (1978) for a more detailed discussion on the local nature of Gorman Polar Form preferences.

4
Varian (1982) showed that any data set $\left\{ \mathbf {p}_{t},\mathbf {q} _{t}^{h}\right\} _{t\in \tau }$ that can be rationalised by a well-behaved utility function will also satisfy the *Generalized Axiom of Revealed Preference* (GARP). We opted to keep our statement of Afriat’s Theorem in line with our following discussion, which will focus on Afriat inequalities.

5 An alternative revealed preference characterisation of the Gorman Polar Form can be found in work in progress by Brown and Shannon. In a certain sense, the work of these authors is complementary to ours as Brown and Shannon characterise Gorman Polar Form preferences in terms of so-called ‘dual’ Afriat numbers (which have an interpretation in terms of indirect utility functions; see Brown and Shannon 2000), whereas our analysis starts from the original ‘primal’ Afriat numbers (to be interpreted in terms of direct utility functions). We thank Don Brown for revealing this to us in a private conversation.

6 Given that $w^h_t\in \mathbb {R}_+$ and $b_t^h \in \mathbb {R}_{++}$, condition (2.*B*.2) implies that $\mathbf {p}_t^{\prime }\mathbf {q}_t \ge a^h_t$. This is intuitive if one wants to interpret $a^h_t$ as a subsistence expenditure.

7 Specifically, under nonproportional price movements, condition (2.*B*.3) becomes redundant. Then, one can easily verify that, for any given solution for the Afriat inequalities $\left( 2.B.1\right)$, there also exists a solution for condition $\left( 2.B.2\right)$.

8 At this point it is worth recalling that we focus on preferences taking the Gorman Polar Form for income values within bounded ranges, which here means that this equivalence has a local nature by construction. The bounds on the income values can be determined by a standard sensitivity analysis of linear programming results (see Appendix 1 for a more detailed discussion).

9 Thus, our empirical analysis uses households/families as consumers. As Samuelson (1947, p. 224) pointed out, “Attention should also be called to the fact that even the classical economist does not literally have the individual in mind, so much as the family; of course, some hardy souls will pursue the will-o’-the-wisp of sovereignty within the family so as to reduce even these collective indifference curves to an individualistic basis”. See Cherchye, De Rock and Vermeulen (2007, 2011) for a revealed preference characterisation of Chiappori’s (1988) model of collective household consumption that starts from an individualistic basis.

10 Relaxing this assumption to allow for changing preferences is not straightforward. Basically, the revealed preference characterization of exact linear aggregation under changing preferences obtains a set of nonlinear Afriat inequalities, which is difficult to implement empirically. Of course, an easy to implement necessary test for stable preferences over subperiods (and thus changing preferences over the whole period) consists of partitioning the set of observations in correspondingly defined subsets, and test our original revealed preference characterization on these smaller sets.

11 Household preferences over these commodity groups are assumed to be stable over time and separable from durable goods. The commodity groups are (1) food and non-alcoholic drinks at home; (2) alcohol; (3) tobacco; (4) energy at home (heating by electricity); (5) services at home (heating: not electricity, water, furniture repair); (6) nondurables at home (cleaning products); (7) non-durable medicines; (8) medical services; (9) transportation; (10) petrol; (11) leisure (cinema, theatre, clubs for sports); (12) personal services; (13) personal nondurables (toothpaste, soap); (14) restaurants and bars and (15) travelling (holiday).

12 We recall from our discussion in the beginning of Sect.  2 that this equivalence is “local” as it only holds for sufficiently small changes of the individuals’ incomes. In the proof of Theorem 2, we indicate that corresponding income bounds can be obtained by standard methods for sensitivity analysis of linear programs. For compactness, we do not pursue this here, also because our main focus is on checking behavioural consistency with exact linear aggregation, and our following investigation will show that such exact aggregation is rejected even when (only) using our local characterization of Gorman Polar Form preferences.

13 An alternative would be to impose rationalisability on them. Blundell et al. (2008) describe a way to do this. In this case, and in view of the very small number of such households, we opted for simplicity.

14 Notwithstanding the shift towards heterogeneous agents models, it is fair to say the representative consumer still plays an important role in a number of modern macroeconomic models and in macroeconomic textbooks (see, for example, Clarida et al. 1999; Woodford 2003; Uhlig 2010, and Gourio 2012).

15 See, for example, Dow and Werlang (1988), Kirman (1992) and Jerison (1994) for more discussion on representative agents and how this relates to exact linear aggregation. Note that the quest for a normatively significant representative consumer is different from that to a positive representative consumer. In Maliar and Maliar (2003), for example, conditions are derived that allow to describe the aggregate behaviour of heterogeneous consumers by means of a representative consumer who is faced with preference, technology and labor shocks. Although this approach allows to make predictions with respect to the aggregate economy in an easier way, the underlying ‘social welfare function’ that represents the preferences of the ‘representative’ consumer is not normatively significant.
